# A Novel Step–Doped Channel AlGaN/GaN HEMTs with Improved Breakdown Performance

**DOI:** 10.3390/mi12101244

**Published:** 2021-10-14

**Authors:** Jianhua Liu, Yufeng Guo, Jun Zhang, Jiafei Yao, Man Li, Maolin Zhang, Jing Chen, Xiaoming Huang, Chenyang Huang

**Affiliations:** 1 College of Electronic and Optical Engineering and College of Microelectronics, Nanjing University of Posts and Telecommunications, Nanjing 210023, China; jhliu_njupt@163.com (J.L.); jfyao@njupt.edu.cn (J.Y.); qiqing0206@163.com (M.L.); zhangml5277@163.com (M.Z.); cjjcnjupt@163.com (J.C.); huangxm@njupt.edu.cn (X.H.); hcy611@126.com (C.H.); 2 National and Local Joint Engineering Laboratory of RF Integration and Micro-Assembly Technology, Nanjing University of Posts and Telecommunications, Nanjing 210023, China

**Keywords:** AlGaN/GaN, high electron mobility transistor (HEMT), step–doped channel, analytical model, electric field (E–field) distribution, breakdown voltage (BV)

## Abstract

The AlGaN/GaN high electron mobility transistor with a step–doped channel (SDC–HEMT) is first proposed in this paper. The potential distribution and the electric field (E–field) distribution of the device are explored by the numerical approach and analytical approach simultaneously. By introducing extra dopants to the channel layer, the E–field distribution along the AlGaN/GaN heterojunction interface is reshaped, resulting in an improved breakdown characteristic. An optimized doping concentration gradient of channel layer of 2 × 10^16^ cm^−3^/step is proposed and verified by simulations. The breakdown voltage (BV) of the optimized SDC–HEMT reaches 1486 V with a 59.8% improvement compared with conventional AlGaN/GaN HEMT. In addition, the average E–field in the region between gate and drain improves from 1.5 to 2.5 MV/cm. Based on the equivalent potential method (EPM), an analytical model of the E–field and potential distribution is presented. The veracity and effectiveness of the proposed methodology is verified by the good agreement between the simulated and modeled results.

## 1. Introduction

The GaN and corresponding semiconductor alloys are recognized as the candidate material for the third–generation power devices owing to the wide bandgap and high electron saturation mobility. Besides the inherent material properties of GaN, the formation of the AlGaN/GaN heterojunction contributes to the superior performance of GaN–based power devices compared with the Si or GaAs–based counterparts [1,2]. Profiting from the polarization induced two–dimension electron gas (2–DEG) at the heterojunction interface, the typical GaN–based power device, HEMT, could achieve high breakdown voltage (BV) and low specific on–resistance (*R*_on,sp_) simultaneously. Intensive investigations have been implemented to push the boundary of the HEMT performance in power applications, such as the power supply, motor drive, PV inverter, and so on [3,4,5,6,7]. In addition, BV is reckoned as the vital parameter to evaluate its power handling capacity. To pursuing better performance, great efforts have been devoted to optimizing the process of film epitaxy and device fabrication. Moreover, structure engineering is regarded as an effective approach to obtain higher BV. Therefore, a more direct approach in improving the device’s BV characteristic is the uniformity of the electric field in off–state. It has been widely recognized that the nonuniformly distributed E–field in the HEMTs plays a decisive role in the pre–mature breakdown of the device. In light of that, the field plate technique is employed in GaN–based HEMT to curb the severe electric field crowding near gate and drain electrodes. As one of the classic junction terminal techniques, the field plates are efficient to transfer E–field peaks but can hardly create an even electric field distribution of the channel layer between the gate and drain [8,9,10,11,12].

In this work, we demonstrate the HEMT with the step–doped channel (SDC–HEMT) to modulate the E–field distribution, thus the more uniform the E–field profile and better breakdown performance could be obtained. The BV could reach 1486 V with 59.8% improvement compared with the conventional device structure and the average E–field between the gate and drain could reach 2.5 MV/cm. Considering that the numerical simulation is time–consuming and the convergence problem is severe for wide–bandgap semiconductor devices, especially for HEMTs with multiple layers involving complicated traps, we proposed a simple and accurate analytical model for SDC–HEMT to analyze the potential distribution and the E–field distribution at the heterojunction interface. The veracity and simplicity of the proposed model are verified by the good agreement between analytical results and simulation results obtained by Sentaurus.

## 2. Numerical Simulation of SDC–HEMT

**Figure 1:**
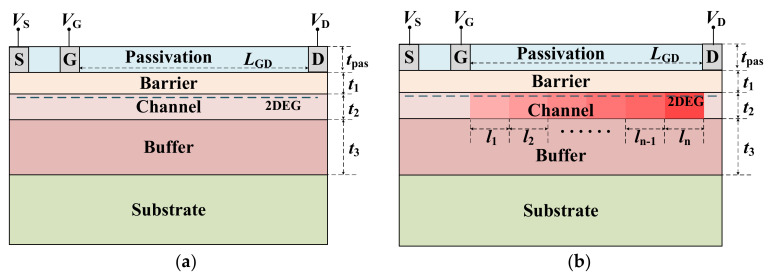
Schematic of the cross–section of (**a**) the conventional HEMT and (**b**) the *n*–step SDC–HEMT.

According to Poisson’s equation, the maximum breakdown voltage of a lateral structure is achieved when an even lateral electric field is reached. In this case, with a particular gate to drain distance, our main concern is therefore the average E–field. Herein, the step doping technology is employed in the structure engineering of HEMT. Figure 1 shows the cross–section of the conventional HEMT and the *n*–step SDC–HEMT. By introducing nonuniform dopants in the channel layer, the distribution of the fixed charges in the depletion region would be reshaped when the device is reversed–biased (*V*_S_ = 0 V, *V*_G_ = −7 V, *V*_D_ > 0 V). The reshaped charge distribution leads to the modulation of the E–field profile at the heterojunction interface. As shown in Figure 1, the *V*_S_, *V*_G_, and *V*_D_ are the voltages applied at the source, gate, and drain electrodes, respectively. The gate–to–drain distance is denoted as *L*_GD_ and the length of each step–doped region (*l*_1_ to *l_n_*) is set as 1 μm. In the vertical direction, the thicknesses of the passivation layer (*t*_pas_), barrier layer (*t*_1_), channel layer (*t*_2_), and buffer layer (*t*_3_) are set as 200 nm, 20 nm, 1 μm, and 3 μm, respectively. The Al composition (*x*) is set as 0.2, and the intrinsic background carrier concentration of the GaN and AlGaN (*N*_1_) is set as 1×10^15^ cm^−3^. The concentrations of the step–doped regions are denoted as *N*_2,*j*_ (*j* = 1 to *n*), respectively, which have taken the intrinsic background carrier concentration into consideration. Here, *N*_2,*j+*1_ = *N*_2,*j*_ + Δ*N* (*j* = 1 to *n* − 1), and Δ*N* is the doping concentration gradient of the step–doped channel. The basic semiconductor equations, such as Poisson, drift–diffusion, and current–continuity equations, are included in the numerical simulation. The physical parameter models contain Shockley–Read–Hall for recombination, impact ionization for generation, high field–dependent mobility model, polarization model, carrier statistic model, and tunneling at the ohmic contacts.

**Figure 2:**
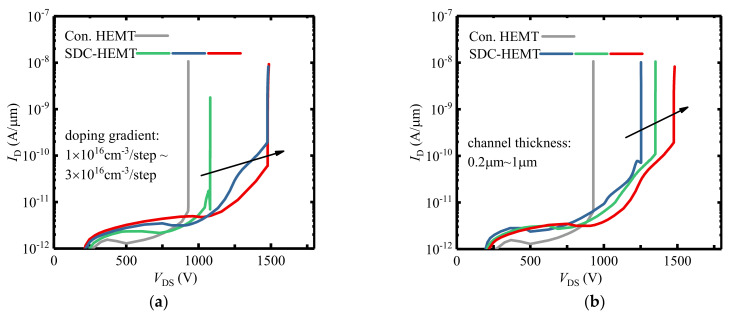
The off–sate *I*_d_–*V*_d_ characteristics of the conventional HEMT and the SDC–HEMTs with (**a**) various doping gradients (the channel thicknesses of SDC–HEMTs are 1 μm) and (**b**) various channel thicknesses (the doping gradients of the SDC–HEMTs are 2 × 10^16^ cm^−3^/step). The channel thickness of the conventional HEMT is 1 μm.

In SDC–HEMTs, the charge distribution of the channel layer is vital to the improvement of the device’s breakdown characteristic. Same as that in Si–based lateral power devices, the depletion region doping dose (*N*(*x*) × *t*) of the proposed SDC–HEMT has a decisive impact on the E–field distribution. Various doping concentration gradients are explored using TCAD tools. Here, the explored devices are six–step SDC–HEMTs with *n* set as 6. Figure 2a illustrates the off–sate *I*_d_–*V*_d_ characteristics of the conventional HEMT and the SDC–HEMTs with a different Δ*N*. The BV of the conventional HEMT is 930 V, and the BV of SDC–HEMT is enhanced significantly with the step doping technology employed. Moreover, the BV of the SDC–HEMT is improved with increased Δ*N*. The improvement would saturate when Δ*N* reaches 2 × 10^16^ cm^−3^/step with BV reaching 1486 V. Except for the doping concentration profile, the doping dose is influenced by the channel thickness as well. To investigate the breakdown of SDC–HEMTs with various channel thicknesses, the doping concentration gradient is set as 2 × 10^16^ cm^−3^/step, and the channel thickness varies between 0.2 μm and 1 μm. Figure 2b demonstrates the off–sate *I*_d_–*V*_d_ characteristics of the conventional HEMT and the SDC–HEMTs with different channel thicknesses. The SDC–HEMTs could achieve higher BV compared with the conventional HEMT. In addition, the BV of SDC–HEMT is enhanced with the thicker channel layer, with 34.7%, 45.3%, 59.4% improvement for the channel thicknesses set as 0.2 μm, 0.6 μm, and 1 μm, respectively. This indicates that the thicker step–doped channel results in higher dopant dose, thus having more significant impacts on BV improvement.

**Figure 3:**
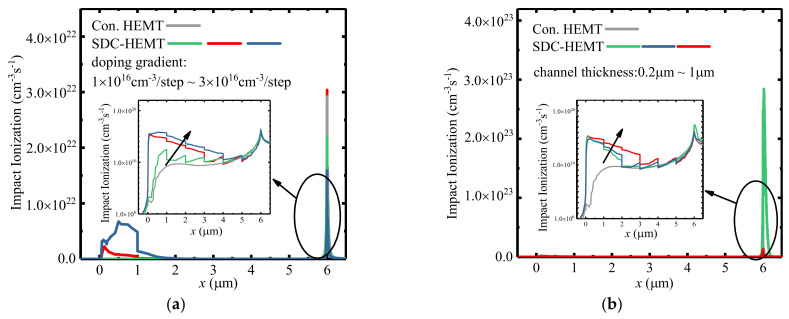
The impact ionization rate distribution at the heterojunction interface of the SDC–HEMTs with (**a**) different doping gradients (the channel thicknesses of SDC–HEMTs are 1 μm) and (**b**) different channel thicknesses (the doping gradients of the SDC–HEMTs are 2 × 10^16^ cm^−3^/step) when the breakdown occurs. The channel thickness of the conventional HEMT is 1 μm.

The impact ionization rate is the direct indicator to evaluate the breakdown position of SDC–HEMT. Figure 3 illustrates the impact ionization rate distribution at the AlGaN/GaN heterojunction interface of the conventional HEMT and SDC–HEMTs when the breakdown occurs. Obviously, a high E–field peak at the drain electrode leads to a remarkable impact ionization rate resulting in the devices’ avalanche breakdown. As shown in Figure 3a, the impact ionization rate near the gate electrode is enhanced with the increased doping gradient. This is why the impact ionization rate is determined by the ionization coefficient and carrier density simultaneously. In addition, the ionization coefficient has a strong relationship with the E–field. The carrier density is higher in the region with higher doping concentration when the device sustains high drain voltage. With an increased doping gradient, the region near the gate exists at a higher carrier density and higher E–field, thus contributing to higher impact ionization. The E–field at the drain electrode decreases with an increased doping gradient, yet the carrier density increased with increased doping gradient. Hence, the impact ionization at the drain electrode is not monotonously decreased with the increased doping gradient. This results in a more uniform impact ionization rate distribution, namely, a higher BV. In addition, as shown in Figure 3b, the impact ionization rate of SDC–HEMTs at the drain electrode increased dramatically with a thinner channel layer, which tends to trigger the breakdown at the drain electrode. The BV of SDC–HEMTs would decrease with a thinner channel layer. With the employment of the step–doped channel, the impact ionization rate distribution could be reshaped to a more uniform state, thus leading to better breakdown characteristics. Although a high E–field peak may exist near the gate electrode, the impact ionization rate near the gate electrode could be neglectable compared with the impact ionization rate near the drain electrode as shown in Figure 3. Thus, breakdown tends to occur at the drain electrode. This is why the region near the drain electrode is heavily doped to emulate the formation of ohmic contact. Impact ionization tends to be triggered in the heavily doped region with massive electrons, thus leading to device breakdown. Therefore, the more uniform distributed impact ionization rate could be achieved by the step doping technology, thus contributing to higher BV.

**Figure 4:**
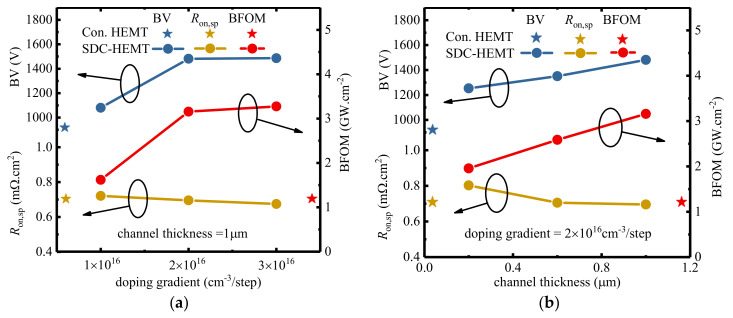
The BV, *R*_on,sp_, and BFOM of the SDC–HEMTs with (**a**) various doping gradients and (**b**) various channel thicknesses.

Apart from the voltage handling capacity improvement that the SDC–HEMT offers, the additionally introduced charges in the step–doped channel could also provide an extra current handling capacity, namely, a lowered *R*_on,sp_. Figure 4 illustrates the BV, *R*_on,sp_, and BFOM of the SDC–HEMTs with various doping gradients and channel thicknesses. As shown in Figure 4a, the *R*_on,sp_ of the SDC–HEMT is slightly decreased with the increased doping gradient, from 0.72 mΩ·cm^2^ to 0.67 mΩ·cm^2^, achieving 7.0% improvement. Meanwhile, the BV is enhanced with the increased doping gradient, contributing to significant BFOM improvement. Notice the BFOM saturate when Δ*N* reaches 2 × 10^16^ cm^−3^/step. In addition,, overhigh Δ*N* would trigger the partially depleted condition resulting in a significant degradation in the breakdown characteristic. Hence, as shown in Figure 4a, the doping gradient of 2 × 10^16^ cm^−3^/step is the optimized value for the device investigated. Figure 4b demonstrates that the SDC–HEMT with a thicker channel layer could achieve lower *R*_on,sp_, from 0.80 mΩ·cm^2^ to 0.70 mΩ·cm^2^, with 12.5% improvement. In accordance with the BV improvement, BFOM would be enhanced with a thicker channel layer with enhancement reaching 259% compared to the conventional HEMT (1.22 GW·cm^−2^). When the channel is too thick, the leakage current in the channel layer would result in a dramatic decrease of BV. This is owing to the intrinsic background carrier that existed in the GaN channel layer. Thus, the optimized channel thickness is 1 μm to achieve satisfying performance.

## 3. Analytical Model of SDC–HEMT

**Figure 5:**
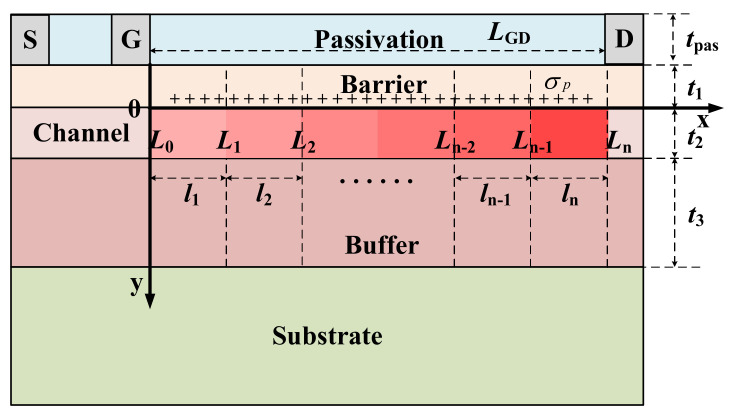
Schematic of the modeling architecture of the *n*–step SDC–HEMT. The region between gate and drain is fully depleted in off–state.

By employing step doping technology, a more uniform impact ionization rate distribution is obtained, leading to improved BV. The reshaped impact ionization rate distribution also results from the E–field modulation effect. To explore the breakdown characteristic of SDC–HEMT, the potential distribution and the E–field distribution at the heterojunction interface deserve intensive investigations. The analytical model is demonstrated to analyze the potential distribution and the E–field distribution of SDC–HEMT. When the SDC–HEMT is reversed–biased with substrate floating, the buffer layer, the channel layer, and the barrier layer are depleted. The buffer layer could be equivalent to a p–type semiconductor mathematically, considering the contribution of acceptor–like traps [13]. The potential distribution could be obtained by solving 2D Poisson equations. The devices explored in this work operate in a fully depleted condition to obtain better breakdown characteristics. Figure 5 shows the schematic of the modeling architecture of the *n*–step SDC–HEMT. The depletion region could be split into *n* segments in the lateral direction mathematically. Here, the position at the cross–point of the heterojunction interface and the right edge of the gate electrode is set as zero. The *x*–axis indicates the lateral distance relative to the right edge of the gate electrode and the *y*–axis indicates the vertical distance relative to the heterojunction interface. The coordinates of the boundaries of the segments are denoted as *L_j_* (*j* = 0 to *n*), respectively, where *L*_0_ = 0 and *L*_n_ = *L*_GD_. For the convenience of modeling, the potential in the depletion region is denoted as *φ_i_*_,*j*_ (*x*, *y*) (*L_j_*_−1_ < *x* < *L_j_*, *d*_i−1_ < *y* < *d*_i_, *i* = 1, 2, 3, *j* = 1 to *n*). And *d*_0_ = −*t*_1_, *d*_1_ = 0, *d*_2_ = *t*_2_, *d*_3_ = *t*_2_ + *t*_3_. The 2–D potential functions could be obtained by solving Poisson equations:

(1) $$\frac{\partial^{2}\varphi_{i,j}\left(x,y\right)}{\partial x^{2}}+\frac{\partial^{2}\varphi_{i,j}\left(x,y\right)}{\partial y^{2}}=\frac{qN_{i,j}}{\varepsilon_{2}},j=1,2,3j=1,\ldots ,n$$

Various charge quantities at the right–hand side of Equation (1) are utilized in the iteration, resulting in the complexity of the modeling process. Thus, the EPM is employed to simplify the modeling approach [14]. The depletion region is divided into two parts along the AlGaN/GaN heterojunction interface. The region above the interface is defined as the top region, and the region below the interface is defined as the bottom region. By employing EPM, charges in the depletion region can be equivalent to the potentials at the passivation surface and the bottom of the buffer as boundary conditions denoted as *V*_top_ and *V*_bot_, respectively. Thus, the depletion region could be assumed as a neutral semiconductor mathematically. The Poisson equations could degenerate into the Laplace equation as Equation (2). Here, the channel layer and the buffer layer of the SDC–HEMT are both GaN material. Therefore, the two layers could be reckoned as an integral layer for simplicity. The 2–D potential function in the integral layer is denoted as *φ*_4,*j*_ (*x*, *y*) (*L_j_*_−1_ < *x* < *L_j_*, *d*_1_ < *y* < *d*_3_, *j* = 1 to *n*). *V*_top_ and *V*_bot_ are expressed in the Appendix A.

(2) $$\frac{\partial^{2}\varphi_{i,j}\left(x,y\right)}{\partial x^{2}}+\frac{\partial^{2}\varphi_{i,j}\left(x,y\right)}{\partial y^{2}}=0,i=1,4j=1,\ldots ,n$$

The potential distribution at the AlGaN/GaN interface is expressed as *φ_j_* (*x*, 0) = *φ*_1, *j*_ (*x*, 0) = *φ*_4,*j*_ (*x*, 0), *j* = 1 to *n*. By substituting the boundary conditions (Equations (3)–(6)) into Equation (2) at *y* = 0, the potential distribution at the interface could be solved as Equation (7). The parameter *T_j_* is the characteristic thickness of SDC–HEMT, which is determined by the device structure. Here, *γ_j_* is the correction factor to take the curvature effect around the electrodes into account. The parameters *V*_0, *j*_, *V*_1,*j*_, *V*_2,*j*_, and *T_j_* (*j* = 1 to *n*) are expressed in Appendix A.

(3) $$-\varepsilon_{1}\frac{\partial \varphi_{1,j}\left(x,y\right)}{\partial y}{|}_{y=d_{0}}=\varepsilon_{pas}\frac{V_{\mathrm{top}}}{t_{pas}}.-\varepsilon_{1}\frac{\partial \varphi_{1,j}\left(x,y\right)}{\partial y}{|}_{y=0}=-\varepsilon_{2}\frac{\partial \varphi_{4,j}\left(x,y\right)}{\partial y}{|}_{y=0}.j=1,\ldots ,n$$

(4) $$\varphi_{1,j}\left(x,0\right)=\varphi_{4,j}\left(x,0\right),\varphi_{4,j}(x,d)=V_{bot,j}+V_{ref},\frac{\partial^{2}\varphi_{1,j}\left(x,y\right)}{\partial y^{2}}{|}_{y=0}=\frac{\partial^{2}\varphi_{4,j}\left(x,y\right)}{\partial y^{2}}{|}_{y=0},j=1,\ldots ,n$$

(5) $$\varphi_{1}\left(L_{0},0\right)=V_{G},\varphi_{n}\left(L_{n},0\right)=V_{D}$$

(6) $$\frac{\partial \varphi_{j}\left(x,0\right)}{\partial x}{|}_{x=L_{j}}=\frac{\partial \varphi_{j+1}\left(x,0\right)}{\partial x}{|}_{x=L_{j}},\varphi_{j}\left(L_{j},0\right)=\varphi_{j+1}\left(L_{j},0\right),j=1,\ldots ,n-1$$

(7) $$\varphi_{j}\left(x,0\right)=V_{1,j}\sinh\frac{x-L_{j-1}}{T_{j}}+V_{2,j}\sinh\frac{L_{j}-x}{T_{j}}+V_{0,j},L_{j-1}\leq x<L_{j},j=1,\ldots ,n$$

Moreover, the E–field at the heterojunction interface could be obtained by the derivation of Equation (7), yielding as Equation (8).

(8) $$E_{j}\left(x,0\right)=\frac{V_{1,j}}{T_{j}}\cosh\frac{x-L_{j-1}}{T_{j}}-\frac{V_{2,j}}{T_{j}}\cosh\frac{L_{j}-x}{T_{j}},L_{j-1}\leq x<L_{j},j=1,\ldots ,n$$

**Figure 6:**
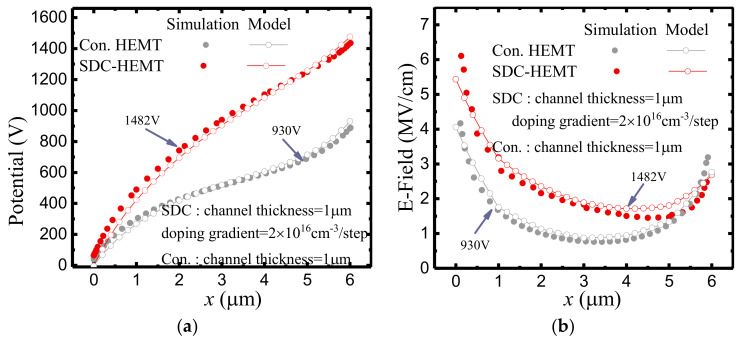
(**a**) The potential distributions and (**b**) the E–field distributions of the conventional HEMT and the SDC–HEMT.

So far, the E–field distribution and the potential distribution at the AlGaN/GaN heterojunction interface have been derived. As shown in Figure 6, the peak–valley structure of the E–field resulted in a limited BV, since the E–field valley can hardly contribute to the device’s voltage handling capacity. Thus, the structure–dependent E–field and potential distribution ought to be further investigated in order to realize a higher BV. Figure 6 demonstrates the potential distributions and the E–field distributions of the conventional HEMT and the SDC–HEMT. The analytical results are in good agreement with the simulation results. With the employment of the step doping technology, the E–field valley is enhanced significantly, and the BV of the HEMT is enhanced from 930 V to 1482 V.

**Figure 7:**
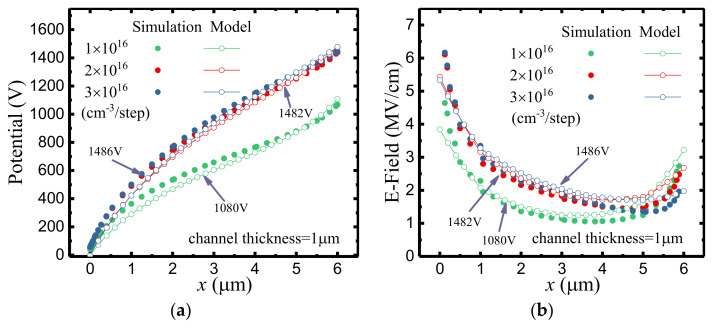
(**a**) The potential distributions and (**b**) the E–field distributions of SDC–HEMTs with different doping gradients.

**Figure 8:**
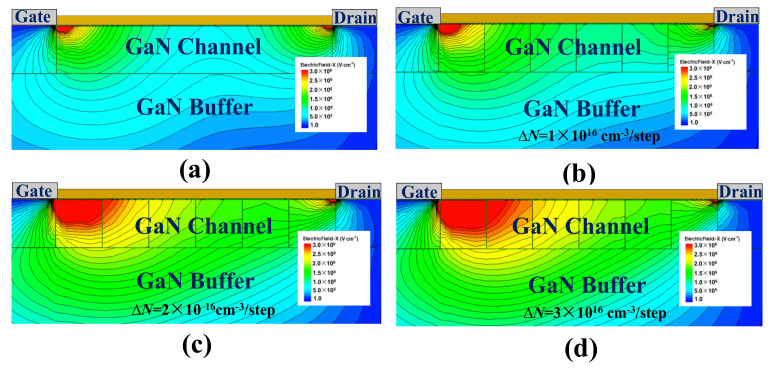
The 2–D E–field distributions of (**a**) the conventional HEMT and (**b**–**d**) the SDC–HEMTs with different doping gradients.

Figure 7 illustrates the potential distributions and the E–field distributions of SDC–HEMTs under various doping doses. With the employment of step doping technology, as shown in Figure 7a, the BV exhibits a remarkable improvement with a much smoother potential distribution along the depletion region. This is consistent with the E–field distribution demonstrated in Figure 7b. With an increased doping gradient, the variation of the doping dose becomes more obvious. Therefore, the valley of the E–field is lifted. Considering the fact that the BV of the device is the integral of the lateral E–field, the enhancement of the E–field valley leads to a higher BV with the same *L*_GD_. As shown in Figure 8, the 2–D E–field distribution is modulated with the employment of the step–doped channel layer, the increased doping gradient contributing to the more uniform E–field distribution.

**Figure 9:**
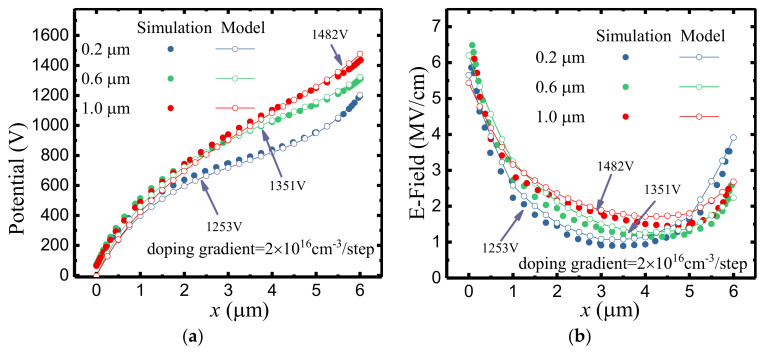
(**a**) The potential distributions and (**b**) the E–field distributions of SDC–HEMTs with different channel thicknesses.

**Figure 10:**
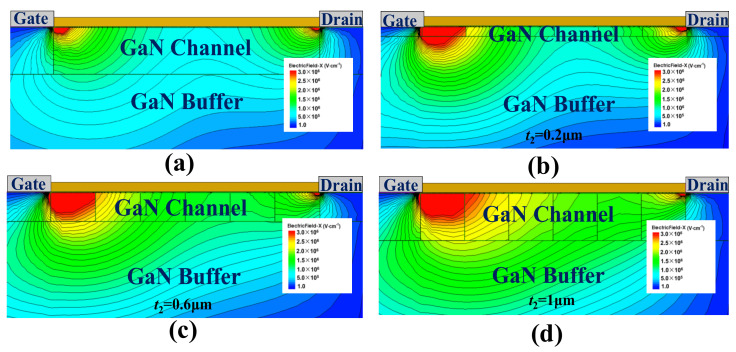
The 2–D E–field distributions of (**a**) the conventional HEMT and (**b**–**d**) the SDC–HEMTs with different channel thicknesses.

Moreover, Figure 9 shows the impact of channel layer thickness on the device’s E–field and potential distribution. As Figure 9a indicates, the potential distribution becomes smoother with a thicker channel layer. From the perspective of the E–field distribution, as Figure 9b shows, the value of the E–field valley is enhanced with the thicker channel layer contributing to the more uniform E–field distribution. This is why the more significant variation of the doping dose could influence the E–field distribution more dramatically. This is consistent with the 2–D E–field distribution of SDC–HEMT. As shown in Figure 10, the contours of the E–field become uniform with a thicker channel layer contributing to a higher BV.

## 4. Conclusions

In this paper, a novel SDC–HEMT is proposed to even the E–field distribution by introducing step–doped dopants in the channel layer. Based on the SDC–HEMT, an analytical model of the E–field and potential distribution is presented accordingly. The E–field distribution of the SDC–HEMT is investigated by the numerical and analytical approaches. The veracity and effectiveness of the proposed method are well verified by the good agreement between the numerical and modeling results. According to this research, the doping dose has proven to be essential to the E–field distribution of SDC–HEMT. An optimized guideline for the doping dose designing is obtained and verified. The BV of SDC–HEMT improved 59.8% from 930 V to 1486 V with the average E–field between the gate and drain reaching 2.5 MV/cm.

## Data Availability

Not applicable.

## Appendix A

$$V_{top}=q\sigma_{p}\left(\frac{t_{1}}{\varepsilon_{1}}+\frac{t_{pas}}{\varepsilon_{pas}}\right)+qN_{1}\left(\frac{t_{1}{}^{2}}{2\varepsilon_{1}}+\frac{t_{1}t_{pas}}{\varepsilon_{pas}}\right),V_{bot,j}=qN_{2,j}\left(\frac{t_{2}{}^{2}}{2\varepsilon_{2}}+\frac{t_{2}t_{3}}{{}_{2}}\right)-qP_{j}\frac{t_{3}{}^{2}}{2\varepsilon_{2}},j=1,\ldots ,n$$

$$T_{j}=\gamma_{j}\left(d_{3}{}^{2}/2+\varepsilon_{1}t_{1}d_{3}/\varepsilon_{2}\right)j=1,\ldots ,n$$

$$V_{0,j}=\frac{\varepsilon_{pas}d}{t_{pas}\varepsilon_{2}}V_{top}+V_{bot,j}+V_{ref},j=1,\ldots ,n$$

$$A_{n}={\left[\begin{array}{ccccc} a_{1} & 1 & & & \\ 1 & a_{2} & 1 & & \\ & \ddots & \ddots & \ddots & \\ & & 1 & a_{n-1} & 1\\ & & & & a_{n} \end{array}\right]}_{n\times n},V_{n}=\left[\begin{array}{c} V_{1,1}\\ V_{1,2}\\ \vdots \\ V_{1,n-1}\\ V_{1,n} \end{array}\right],B_{n}=\left[\begin{array}{c} V_{1}\\ V_{2}\\ \vdots \\ V_{n-1}\\ V_{n} \end{array}\right]$$

$$V_{n}={\left|A_{n}\right|}^{-1}B_{n}$$

$$a_{1}=-\cosh\left(l/T\right)/\gamma_{1}-\sinh\left(l/\gamma_{1}T\right)/\tanh\left(l/T\right),a_{n}=\sinh\left(l/\gamma_{n}T\right)$$

$$V_{1}=\left(V_{0,1}-V_{0,2}\right)/\tanh(l/T)-\left(V_{G}-V_{0,1}\right)/\gamma_{1}\sinh(l/\gamma_{1}T),V_{n}=V_{D}-V_{0,n}$$

$$a_{j}=-2\cosh\left(l/T\right),V_{j-1}=\frac{\left(V_{0,j}-V_{0,j-1}\right)+\cosh\left(l/T\right)\left(V_{0,j}-V_{0,j+1}\right)}{\sinh\left(l/T\right)},j=1,\ldots ,n-1$$

$$V_{2,j}=\left(V_{1,j-1}\sinh\left(l/T_{j-1}\right)+\left(V_{0,j-1}-V_{0,j}\right)\right)/\sinh\left(l/T_{j-1}\right),j=2,\cdots ,n$$

$$V_{2,1}=\left(V_{G}-V_{0,1}\right)/\sinh\left(l_{1}/T_{1}\right)$$
